# Reliability and Validity of the Brief Illness Perception Questionnaire in Bahasa Malaysia for Patients with Cancer

**DOI:** 10.31557/APJCP.2021.22.8.2487

**Published:** 2021-08

**Authors:** Harenthri Devy Alagir Rajah, Chie Qiu Ting, Mahadir Ahmad, Wun Chin Leong, Nirmala Bhoo-Pathy, Caryn Mei Hsien Chan

**Affiliations:** 1 *Faculty of Health Science, Universiti Kebangsaan Malaysia, Campus Kuala Lumpur, Kuala Lumpur, Malaysia.*; 2 *Faculty of Arts and Social Science, University Tunku Abdul Rahman, Kampar, Perak, Malaysia. *; 3 *Faculty of Medicine, Universiti Malaya, Kuala Lumpur, Malaysia. *; 4 *Department of Radiotherapy and Oncology, National Cancer Institute, Putrajaya, Malaysia. *

**Keywords:** Perception of illness, newly diagnosed cancer, psychometric properties

## Abstract

**Objective:**

The main purpose of this study was to identify the psychometric properties and validate the Bahasa Malaysia version of the Brief Illness Perception Questionnaire among patients with cancer.

**Methods:**

A total of 129 newly diagnosed patients with cancer were consecutively sampled. Reliability and validity of the questionnaire were tested using translation validity, test-retest reliability, Principal Component Analysis, Cronbach’s alpha coefficient for domains and item-total correlation.

**Results:**

The questionnaire indicates excellent test-retest reliability. The Principal Component Analysis (PCA) revealed that Kaiser-Meyer-Olkin (KMO) is 0.60 for the two-factor structure of the Brief Illness Perception Questionnaire of the Bahasa Malaysia version which consists of cognitive illness representation and emotional illness representation.

**Conclusion:**

The Brief Illness Perception Questionnaire in the Bahasa Malaysia version is a useful tool to use among patients with cancer in Malaysia context despite moderate psychometric properties. This is based on the premise that the questionnaire can be used as a quick tool to assess illness perceptions among Malaysian with cancer in routine oncology practice.

## Introduction

Cancer is a major lifelong disease and a leading cause of morbidity and mortality worldwide (Chou, 2019). The number of new cancer cases is predicted to rise to over 70% globally in the next two decades with an estimated 10.6 million cases in Asia by 2030 (World Health Organization, 2018). In 2016, cancer was the fourth leading cause of death in Malaysia contributing to 12.6% of overall deaths in the government hospitals and 26.7% in the private hospitals. Negative illness perceptions among cancer patients is a cause of concern as it is associated with higher mortality among cancer survivors. This may be explained by the fact that these perceptions may negatively influence cancer patients’ self-management behavior toward symptoms and treatment (Sawyer et al., 2019). Besides that, there remains strong stigma attached to the perception of cancer as a serious and chronic disease, leading to desperation and uncertainty (Peltzer and Pengpid, 2016). A previous study in Southeast Asia had shown that Indonesian women described breast cancer as a dangerous illness that is fatal and incurable. Notably, the study participants had associated breast cancer diagnosis and its treatment with disability, distortion in physical appearance, and economic ramifications (Iskandarsyah et al., 2013). 

Illness perception refers to a patient’s cognitive appraisal and their understanding of a medical condition and its future consequences (Broadbent et al., 2006). It encompasses both positive and negative illness beliefs which can affect the patient’s ability to cope with the illness, depending on whether the latter is perceived as manageable or threatening (Broadbent et al., 2006). The concept of illness perception originates from the Common Sense Self- Regulation Model of illness representation (Leventhal, 1980). Based on this model, the health outcomes of patients with chronic conditions are influenced by their cognitive and emotional illness representation wherein patients incorporate lay information, personal experience, and information from the social environment to form an interpretation of the impact of the disease on their lives (Hagger et al., 2017). 

The Brief Illness Perception Questionnaire consists of 9 items designed to provide a simple and quick assessment of illness perception. A previous validation study of the Bahasa Malaysia version of the Brief Illness Perception among patients with type 2 diabetes mellitus indicated moderate test-retest reliability and construct validity, and also acceptable cross-cultural validity (Chew et al., 2017). In the present study, we determined the reliability and validity of the Bahasa Malaysia version of the Brief Illness Perception Questionnaire in patients with cancer. This is based on the premise that the questionnaire can be used as a quick tool to assess illness perceptions among Malaysian with cancer in routine oncology practice. 

## Materials and Methods

*Study tool: Brief Illness Perception Questionnaire (BIPQ)*

Brief Illness Perception Questionnaire (BIPQ) is the simplified version of the Illness Perception Questionnaire (IPQ). It comprises three components including cognitive illness representation, emotional illness representation, and illness comprehensibility representation (Broadbent et al., 2006).The first five items assess cognitive representation, specifically perceived consequences (Item 1), timeline (Item 2), personal control (Item 3), treatment control (Item 4), and the presenting symptoms or identity (Item 5). Emotional illness representation is assessed through concern (Item 6) and emotions (Item 8). Only one item assesses illness comprehensibility or coherence of illness (Item 7). The final question (item 9) is in an open-ended format adapted from the Illness Perception Questionnaire, where patients are asked to list down the three main factors that cause their illness. A higher score indicates a stronger perception of illness perception for each dimension. The Brief Illness Perception Questionnaire has been validated in many illnesses such as diabetes, asthma, renal disease, cancer, and also minor illnesses (Broadbent et al., 2015).

*Participants and data collection*

The study population comprise multiethnic patients attending their first consultation visit at three referral oncology centers in the Klang Valley (University Malaya Medical Centre, Kuala Lumpur Hospital, and the National Cancer Institute). All patients who were aged at least 18 years old, diagnosed with any cancer type within 3 months prior to the study, and were able to understand and converse in Bahasa Malaysia were consecutively recruited. Data collection was carried out by trained postgraduate research assistants via face to face interviews.

*Ethical Consideration*

Formal permission was sought to translate and validate the Brief Illness Perception Questionnaire into Bahasa Malaysia. This study received research ethics approval from the Malaysian Research and Ethics Committee (NMRR-19-118-45622). Written informed consent was obtained from all study participants.

*Statistical analysis*

The distribution of frequencies, percentage, means, and standard deviations (SDs) of demographic variables was analyzed by using the IBM SPSS Statistic for Window, Version 20.0. The reliability of the Brief Illness Questionnaire Perception in Bahasa Malaysia version was examined using test-retest reliability, Cronbach’s alpha, and total item correlation. To determine the validity of the Brief Illness Perception Questionnaire, Principal Component Analysis (PCA) was conducted. 

## Results

*Descriptive Analysis*

A total of 129 cancer patients participated in this validation study. Table 1 summarizes the demographic variables and clinical characteristics of participants who participated in this study. The average age of participants was 48.08 ± 12.46. The majority of patients were female 67.2% (n = 86), 71.9% were married with children, 43.4% were employed and 65.1% household income less than 1026 USD. Most patients in this study were diagnosed with breast cancer 33.3% (n = 43), followed by 22.5% (n = 29) gastrointestinal cancer and 14.0% (n = 18) gynecological cancer. 

*Test-retest reliability analysis*

The test-retest reliability of the Brief Illness Perception Questionnaire in the Bahasa Malaysia version was assessed in 30 patients with cancer from the National Cancer Institute. Participants were tested at two time points. The first time point being the patient’s first presentation to the oncology clinic. The second time point was weeks later, where the Brief Illness Perception Questionnaire was administered again to determine its test-retest reliability. The items were found to have good reliability over 2 weeks with a p-value of less than 0.05 and a Pearson correlation coefficient of 0.94. 

*Translation Validity*

The English version of the Brief Illness Perception Questionnaire was translated into Bahasa Malaysia by four independent professional bilinguals including psychologists and linguistic experts. A standard forward and backward translation was carried out and the versions were back-translated into English version by another two independent translators who were blinded to the intent and purpose of the study. The principal purpose of this step was to check that the translated versions would reflect the same content as the original without any errors, omissions, vagueness, or inaccuracies to reduce any cultural and social bias. Through this step, the review panel was able to verify the content validity and finalized a consistent version by modifying or rejecting inappropriate items/words for final consensus. The finalized questionnaires were piloted to ten participants who were not a part of the targeted sample of this study. Following this, the final version of the Brief Illness Perception Questionnaire in the Bahasa Malaysia version was developed to be used for the reliability and validity study with 129 participants.

*Construct Validity*

Principal Component Analysis (PCA) was used to determine the factorial structure of the Brief Illness Perception Questionnaire (BIPQ) in patients with cancer in the Malaysian context. The result of the sampling adequacy measure reported a Kaiser-Meyer Olkin (KMO) value of 0.60. The Kaiser-Meyer Olkin (KMO) should be equal or greater than 0.6 to indicate adequate sample size (Awang et al., 2018). Bartlett’s test of sphericity was statistically significant *x*^2^ (28) = 185.642, p < 0.001, which indicates suitability of data set for factorial analysis. The principal component analysis rotated to the Promax rotation (25) axis and extracted a 2-factor structure which explained 49.293% of the total variance. The first factor was the emotional illness representation which consists of Item 1 (Consequences), Item 2 (Timeline), Item 5 (Identity), Item 8 (Emotions). The loading of the items in the first factor was ranged between 0.633 and 0.814 and can be explained by 27.588 % of the total variance. The second factor was the cognitive illness representation component which consists of Item 3 (Personal Control), Item 4 (Treatment Control), Item 6 (Coherence), and Item 7 (Comprehensibility). The loading of the items in the second factor ranged between are 0.367 and 0.861 and is explained by 21.735% of the total variance. 

*Scree Plot*

The results of factor analysis by Principal Component Analysis (PCA) indicate the Brief Illness Perception Questionnaire in Bahasa Malaysia for patients with cancer comprises 2 factors which are as shown in the scree plot.

*Internal Consistency*

Internal consistency reliability was analyzed to assess the consistency of results across factors within a test. The correlation between the items and Cronbach’s alpha internal consistency coefficient was reviewed to investigate the capability of each item to measure the characteristics that they intend to measure. The corrected total item correlation scores in emotional illness representations ranged between r = 0.387 and r = 0.544 which indicates good internal consistency for all the items in the emotional illness representation. As for cognitive illness representation, the corrected total item correlation scores in was ranged between r = 0.235 and r = 0.503 which showed good internal consistency for Item 6 (Concern) and Item 7 (Coherence) but moderately acceptable internal consistency for Item 3 (Personal Control) and Item 4 (Treatment Control). The reliability testing showed that Cronbach’s alpha is 0.68 and 0.57 respectively for emotional illness representation and cognitive illness representation. The results of the reliability coefficient indicate a satisfactory level of internal consistency for emotional illness representation and moderately acceptable level of internal consistency for cognitive illness representation. 

**Figure 1:**
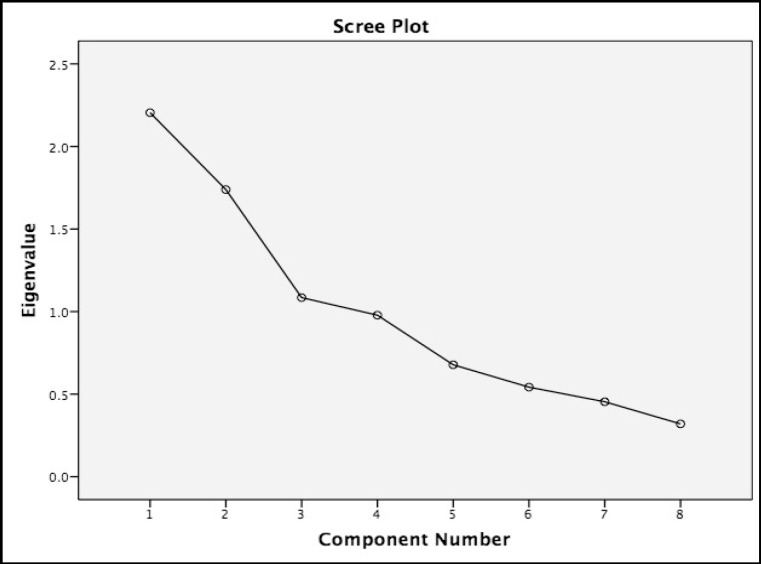
The Brief Illness Perception Questionnaire in Bahasa Malaysia for Patients with Cancer Comprises Two Factors which are as Shown in the Scree Plot

**Table 1:** Characteristics of Participants

Demographic	Overall (N=129) n (%)
Age, years	
18-39	33 (25.6)
40-54	56 (43.4)
55-69	34 (26.4)
70 and above	6 (4.7)
Ethnicity	
Malay	104 (81.3)
Chinese	11 (8.6)
Indian	10 (7.3)
Others	3 (2.3)
Marital status	
Single	13 (10.2)
Single with children	1 (0.8)
Married with children	92 (71.9)
Married with no children	8 (6.3)
Divorced	6 (4.7)
Windowed	8 (6.3)
Unknown	1
Highest education	
Primary	16 (12.5)
Secondary	72 (56.3)
STPM/Matriculation/A-Levels/Diploma	18 (14.1)
Tertiary	21(16.4)
Unknown	2
Monthly Household Income (RM)‡	
B40 (<1026 USD)	82 (65.1)
M40 (1026 USD -2264USD)	24 (19.0)
T20 (>2265 USD)	4 (3.2)
Unknown	19
Employment Status	
Employed	56 (43.8)
Unemployed	43 (33.6)
Retired	20 (15.6)
Others (Housewife)	9 (7.0)
Unknown	1
Type of hospital	
Hospital Kuala Lumpur (HKL)	61 (48.8)
Institute Kanser Negara (IKN)	63 (47.3)
Unive Malay Medical Centre (UMMC)	5 (3.9)
Type of cancer	
Breast	43 (33.3)
Gastrointestinal	29 (22.5)
Gynecological	18 (14.0)
Lung	8 (6.2)
Sarcoma	5 (3.9)
Nose	2 (1.6)
Haemotogical	2 (1.6)
Demographic	Overall (N=129) n (%)
Type of cancer	
Brain	1 (0.8)
Others†	2 (1.6)
Unknown	19(14.7)
Cancer stage	
I	14 (15.1)
II	28 (30.1)
III	26 (28.0)
IV	25 (26.9)

Characteristic of participants were presented in frequencies and percentage; ‡1 MYR, 0.24 USD; † Others type of cancer, Prostate Cancer

**Table 2:** Factors, Factor Loadings and Explained Variances from Principal Component Analysis

Factors and items	Factor loads	Eigenvalue	Variance	Cumulative Variance
Emotional Illness Representation	-	2.205	27.558	27.558
Item 8: How much does your the illness affects you emotionally (e.g. does it make you angry, scared, upset or depressed	0.814	-	-	-
Item 1: How much does your illness affect your life	0.719	-	-	-
Item 5: How much do you experience symptoms from your illness?	0.663	-	-	-
Item 2: How long do you think your illness will continue	0.633	-	-	-
Cognitive Illness Representation	-	1.739	21.735	49.293
Item 7: How well do you feel you understand about your illness?	0.861	-	-	-
Item 6: How concerned are you about your illness?	0.854	-	-	-
Item 3: How much control do you feel you have over your illness?	0.408	-	-	-
Item 4: How much do you think your treatment can help your illness	0.367	-	-	-

**Table 3:** Cronbach’s Alpha and Total Item Correlations

Factors and Items	Scale Mean if Item Deleted	Scale Variance if Item Deleted	Corrected Item-Total Correlation	Cronbach’s Alpha if Item Deleted
Emotional illness representation				
Cronbach’s alpha: 0.68				
Item 8 (Emotions)	11.18	35.968	0.544	0.564
Item 1 (Consequences )	12.02	35.968	0.519	0.582
Item 5 (Identity)	12.76	44.055	0.387	0.655
Item 2 (Timeline)	12.35	42.613	0.419	0.646
Cognitive Illness Representation				
Cronbach’s alpha: 0.57				
Item 7 (Coherence)	21.51	26.173	0.503	0.365
Item 6 (Concern)	21.13	27.738	0.432	0.426
Item 3 (Personal control)	22.5	29.843	0.235	0.596
Item 4 (Treatment control)	20.97	32.235	0.257	0.563

## Discussion

This is the first study validating the Brief Illness Perception Questionnaire (BIPQ) among patients living with cancer in a South East Asian setting. Our study shows that the translated version of the Brief Illness Perception Questionnaire in Bahasa Malaysia can be used as a simple and quick screening tool to evaluate the personal beliefs that patients with cancer hold about their illness. 

Findings from our study indicate a two-factor structure for the Bahasa Malaysia version of the Brief Illness Perception Questionnaire, rather than a three-factor structure, which is in line with the original constructs of Common Sense Self-Regulation Model proposed by Leventhal and colleagues (Leventhal, 1986). This is in contrast to the original Brief Illness Perception Questionnaire, developed by Broadbent and colleagues (Broadbent et al. 2003) which had a three-factor structure whereby the; cognitive illness representation consisted Item 1 (Consequences) Item 2 (Timeline), Item 3 (Personal Control), Item 4 (Treatment Control), and Item 5 (Identity). The emotional illness representation consisted of Item 6 (Concern) and Item 8 (Emotions) while Item 7 (Coherence) falls under illness comprehensibility representation.

The psychometric properties of the Bahasa Malaysia version of the Brief Illness Perception Questionnaire appear similar to the Turkish (Kutluturkan et al., 2017) and Chinese language (Zhang et al., 2017) versions of the questionnaire developed for cancer patients. Both in these language versions were found to have a two-factor structure because the original three-factor structure of cognitive, emotional, and illness comprehensibility fitted poorly the Turkish and Chinese sample. Taken together, these results appear to suggest that factorial structure of the BIPQ is likely to be highly influenced by cultural differences. In other words, participants from different cultural backgrounds seem to generally perceive that the eight items explained three different constructs. This is in line with the school of thought that differing cognitive beliefs and perception of health and illness varies from one culture to another, which can account for differences in patient outcomes (Fischer et al., 2017)

We found that internal consistency to be moderately acceptable for cognitive illness representation in the Bahasa Malaysia version of the Brief Illness Perception Questionnaire. A lower item-total correlation was found for (Personal Control, r = 0.235) and (Treatment Control, r = 0.257) (Nunnally, 1994) emphasized that value greater than 0.30 will be a good indicator that items were related to the overall score. However, deleting these items only marginally increased the Cronbach’s alpha from 0.57 to 0.59. Therefore, the items were retained due to consistency with the original theoretical model 

The current findings were found to be consistent with results in the two-factor model Chinese version of the Brief Illness Perception Questionnaire (Zhang et al., 2017). The internal consistency was also moderately acceptable for the cognitive illness representation in the Chinese version of Brief Illness Perception Questionnaire and also found to have the lowest item-total correlation of r = 0.290 for the Item 3 (Personal Control). The control domain relates to how one perceives the possibility of recovery from or management of a disease that involves both personal control and treatment control (Sawyer et al. 2019). Two prior studies by Kaptien and colleagues among Dutch and Japanese patients with non-small cell lung cancer (Kaptein et al., 2011) and breast cancer (Kaptein et al., 2018) to explore the illness perceptions and quality of life had shown that while the Japanese and Dutch patients shared similar perceptions of cancer as a disease but some differences were noted. Compared with the Dutch patients with non-small cell lung cancer, Japanese patients scored higher on personal control and treatment control, suggesting that the Japanese may have had higher confidence in the success of their cancer treatment. When comparing our results to these previous studies, it must be noted that the majority of patients in the present study were from the lower socioeconomic status. 

Cancer is a major health burden to lower-income groups in Malaysia. Patients from lower-income groups and lower levels of education may feel less control over their illness due to a lower level of health literacy and it is very crucial to increase more specialist oncology healthcare professionals in rural areas to provide cancer care for lower income group and lower literate patients with cancer (Yadav, 2020). For an instance, a previous study found that majority inpatients of late stages (stage III and IV) are from low-income groups due to high risk of catastrophic health costs (Puteh and Almualm, 2017). On the other hand, according to the ASEAN Costs in Oncology (ACTION) study, the large proportion of cancer-stricken households in the low-and –middle –income countries (LMIC) in Southeast Asia endured catastrophic expenditures and poverty in less than 1 year of cancer diagnosis, including Malaysia (Bhoo-Pathy et al., 2019). These factors may hinder patient health-seeking behavior and treatment adherence which directly influence the ability to control over and manage their illness. In line with this, patients with cancer were less likely to know how to reduce the chance of recurrence or having better control over their symptoms (Lambregts, 2017). Last but not least, healthcare professional’s positive attitude regarding cancer illness may influence patient’s cancer management, for an instance, healthcare professional’s positive attitudes are strongly associated with improving patient’s perception and cancer management (Shrestha et al., 2020) 

*Strengths and limitation*

The strength of the present study was its excellent test-retest reliability. Future studies should evaluate the predictive validity and factorial validity of the Bahasa Malaysia version of the Brief Illness Perception questionnaire among cancer patients. Unlike in the past studies, the participants in the present study comprised newly diagnosed cancer patients. It is recognized that the level of perception and ability to manage their illness will change according to the phases of treatment and survivorship. Therefore, further follow-ups to identify the differences in the level of perception from baseline have been planned. 

In conclusion, the present study shows that the two-factor structure of the Bahasa Malaysia version of the Brief Illness Perception Questionnaire is acceptable for use among patients with cancer in the Malaysian context. Despite its moderate psychometric properties, it is a useful tool to enhance and express the personal thoughts and feelings of the patient. 

## Author Contribution Statement

HD recruited the study participants, wrote the manuscript, conducted the analysis and approved final manuscript. CQT supported the data analysis, provide feedback for the manuscript (interpretation of data and revising), approved final manuscript. MA co-supervised the study, supported the data analysis, provide feedback for the manuscript (interpretation of data and revising), approved the final manuscript. WCL recruited the study participants, supported the data analysis and approved final manuscript. NB co-supervised the study, supported the data analysis, provide feedback for the manuscript (interpretation of data and revising), approved final manuscript. CMHC supervised the study, design the study supported the data analysis, provide feedback for the manuscript (interpretation of data and revising), approved final manuscript.

## Funding

The research is funded by Malaysia Research University Network (MRUN)-Long Term Research Grant (LRGS) (NN-2019-090).

## Ethics approval and consent to study participants

The present study was approved by the Malaysian Research and Ethics Committee (NMRR-19-118-45622), Ethics Committee of Universiti Kebangsaan Malaysia (JEP-2020-029) and was conducted according to the Declaration of Helsinki. All the study participants provided written informed consent 

## Statement conflict of interest Disclosure

The authors declare no conflict of interest 
